# Autoría de regalo: elemento para NO considerar en una publicación

**Published:** 2019-12-30

**Authors:** Alejandro Usubillaga-Villa, Herney Andrés García-Perdomo

**Affiliations:** 1 Grupo de Investigación UROGIV, Universidad del Valle, Cali, Colombia Universidad del Valle Grupo de Investigación UROGIV Universidad del Valle Cali Colombia; 2 Departamento de Cirugía/Urología, Escuela de Medicina, Universidad del Valle, Cali, Colombia Universidad del Valle Departamento de Cirugía/Urología Escuela de Medicina Universidad del Valle Cali Colombia

Estimado editor:

Hemos leído con detenimiento el artículo publicado de Zafra-Tanaka y colaboradores ^1^ y hemos encontrado algunos elementos importantes que nos gustaría compartir con los lectores.

Inicialmente, cabe recordar los criterios para definir autoría de artículos científicos médicos acordes con el Comité Internacional de Editores de Revistas Médicas (*International Committee of Medical Journal Editors,* ICMJE) ^2^ y las normas para los autores de la revista *Biomédica* que, aunque no hay una traducción oficial, son claros en su fundamento. Estos son:

- Contribuciones significativas a la concepción o diseño del manuscrito, o la recolección, el análisis o la interpretación de los datos; y
- Redacción del trabajo o revisión crítica del contenido intelectual importante; y
- Aprobación de la versión final para ser publicada; y
- Asumir responsabilidad frente a todos de los aspectos del manuscrito, para garantizar que los asuntos relativos a la exactitud o integridad de cualquier parte del mismo hayan sido apropiadamente investigados y resueltos.

Llama la atención que en el diseño del estudio de Zafra-Tanaka y colaboradores se excluyeron las revisiones narrativas sin justificación alguna, por lo cual se está subestimando la prevalencia de las autorías de regalo.

En el mismo artículo especifican que la prevalencia de autorías de regalo en este tipo de revisiones es del 15 % ^3^, por lo tanto, hubiera sido interesante su búsqueda.

Adicionalmente, en los estudios de Wislar, *et al.*, ^3^ y Mirzazadeh, *et al.*^4^, se usaron cuestionarios enviados a los autores para recolectar información. Preguntaron directamente a cada autor sobre el grado de participación que tuvo cada persona y luego cruzaron la información para comprobar su veracidad. Esto no se contempló en el desarrollo del presente estudio, lo cual sugiere que no hubo una búsqueda activa con los autores del artículo, y sólo corroboraron esa información al revisar los aportes de cada autor, según lo expresaban en el certificado de contribuciones de cada artículo. Esto claramente supondría un sesgo de información que limita los resultados del trabajo publicado.

Para finalizar, aunque es importante investigar las autorías de regalo porque se ha encontrado como una práctica inadecuada y frecuente en los artículos publicados, existe un gran problema fundamental y es que no existe una herramienta estandarizada para auditar la veracidad de la información presentada por cada autor. Por otro lado, sólo quedaría confiar en la integridad científica de los autores cuando realizan sus trabajos de investigación y recomendar a los investigadores jóvenes, el uso estricto de las recomendaciones del ICJME.
